# The dsDNA, Anti-dsDNA Antibody, and Lupus Nephritis: What We Agree on, What Must Be Done, and What the Best Strategy Forward Could Be

**DOI:** 10.3389/fimmu.2019.01104

**Published:** 2019-05-15

**Authors:** Ole Petter Rekvig

**Affiliations:** Department of Medical Biology, Faculty of Health Sciences, University of Tromsø, Tromsø, Norway

**Keywords:** chromatin, dsDNA, autoimmunity, lupus nephritis, enigma, controversies

## Abstract

This study aims to understand what lupus nephritis is, its origin, clinical context, and its pathogenesis. Truly, we encounter many conceptual and immanent tribulations in our attempts to search for the pathogenesis of this disease—and how to explain its assumed link to SLE. Central in the present landscape stay a short history of the early studies that substantiated the structures of isolated or chromatin-assembled mammalian dsDNA, and its assumed, highly controversial role in induction of anti-dsDNA antibodies. Arguments discussed here may provoke the view that anti-dsDNA antibodies are not what we think they are, as they may be antibodies operational in quite different biological contexts, although they bind dsDNA by chance. This may not mean that these antibodies are not pathogenic but they do not inform how they are so. This theoretical study centers the content around the origin and impact of extra-cellular DNA, and if dsDNA has an effect on the adaptive immune system. The pathogenic potential of chromatin-anti-dsDNA antibody interactions is limited to incite lupus nephritis and dermatitis which may be linked in a common pathogenic process. These are major criteria in SLE classification systems but are not shared with other defined manifestations in SLE, which may mean that they are their own disease entities, and not integrated in SLE. Today, the models thought to explain lupus nephritis are divergent and inconsistent. We miss a comprehensive perspective to try the different models against each other. To do this, we need to take all elements of the syndrome SLE into account. This can only be achieved by concentrating on the interactions between autoimmunity, immunopathology, deviant cell death and necrotic chromatin in context of elements of system science. System science provides a framework where data generated by experts can be compared, and tested against each other. This approach open for consensus on central elements making up “lupus nephritis” to separate what we agree on and how to understand the basis for conflicting models. This has not been done yet in a systematic context.

## Introduction

In this critical review, different aspects of pathogenic processes suspected or proven to be involved in lupus nephritis are discussed; (*i)* The exposure of dsDNA, and the impact of its surface structure and net charge exposed in pure dsDNA vs. DNA in chromatin; (*ii)* Anti-dsDNA antibodies, whether homologous or heterologous depending on whether instigated by DNA or non-DNA structures, and what they recognize in glomeruli; (*iii)* If lupus nephritis in a critical sense is an intrinsic part of SLE; and as a direct consequence of the last question; (*iv)* Whether SLE is an abstraction without a clear definition, which may allow us to regard lupus nephritis as a single disease entity; and (*v)* Whether production of anti-dsDNA antibodies induce the same pathogenic processes in non-SLE (like in cancer) patients as they do in SLE. In other words, can lupus nephritis etiologically be regarded as an integrated part of SLE—or can it stand alone? These dilemmas may not center around a clinical diagnosis, but around processes that may describe the molecular and cellular events that in sum define lupus nephritis. In this context, it is important to discuss factors that prime the inflammatory processes in lupus nephritis, and not secondary inflammatory mediators like complement activation, cytokines or their receptors, because the initiators of lupus nephritis inherit the principle, while inflammatory pathways are secondary responses instigated by the principal inducers of lupus nephritis—like type II or type III immune mediated tissue inflammation. In fact, if we summarize data over the last decades, both type II and type III have been claimed to account for lupus nephritis. One tribulation is whether type II immune mediated nephritis is more like Goodpasture syndrome (1, 2) than like lupus nephritis. However, there are many more problems that need to be solved before we can develop a true pathogenic model of lupus nephritis (see below). These problems represent the focus of this study.

## The dsDNA: Structure, Autoimmune Inducer, and Target—Status and a Short Scientific History

In two foregoing studies, an historical and contemporary overview of anti-dsDNA antibodies (3) and a condensed history of the evolution of our contemporary opinions on SLE (4) have been published. These two studies aimed at a central understanding of the role of dsDNA and how it is involved in lupus nephritis. On the other hand, it is possible that dsDNA plays a bystander role in the disease, if e.g., anti-dsDNA antibodies recognize different obligate glomerular structures (see below). In that sense it is essential to approach historical and contemporary studies and hypotheses as backdrops to understand how paradigms related to SLE and anti-dsDNA antibodies have evolved over time. In other words, history is also in this context important to consider in order to understand contemporary paradigms. Ludvik Fleck once said*: “For the current state of knowledge remains vague when history is not considered, just as history remains vague without substantive knowledge of the current state”* [(5), cited in (4)].

Whether the antibodies described in 1957 in SLE (6–9) were specific for dsDNA and not for other DNA structures like ssDNA can be discussed in terms of history of science on dsDNA. The scientific history of DNA originates from studies performed during the 19th century. DNA was first identified as a unique substance in the late 1860s by the Swiss chemist Friedrich Miescher [(10), see also the biographical presentation of Miescher by (11)]. In the aftermath of Miescher's discovery, studies revealed fundamental details about the DNA molecule. This resulted in important discoveries describing the chemical composition of DNA, including its primary chemical components and the ways in which chains joined with one each other. Central scientists were Phoebus Levene, who provided evidence that different forms of nucleic acids existed—DNA and RNA, and he also determined that DNA contained adenine, guanine, thymine, cytosine, deoxyribose, and a phosphate group (12); and Erwin Chargaff, who was the first to present evidence that the DNA structure exists as a double helix constituted by two complementary single-strand DNA molecules (13, 14) (see below). With these important and pioneering studies, the enigma of inheritance started to be revealed.

A central researcher aiming to solve this scientific puzzle was Rosalind Elsie Franklin, a British chemist [see the comprehensive biography on Rosalind Franklin by Brenda Maddox, (15)]. Franklin contributed significantly to the discovery of the three-dimensional structure of dsDNA by X-ray crystallography (16). In these studies, she precisely described the double helix of DNA, a discovery that placed her in the first row of those days biochemical scientists aimed to describe the nature, structure and function, basically of Miescher's “nuclein” (11) and transformed it into dsDNA! Watson and Crick and their studies that were published in 1953 stated that the DNA molecule exists in the form of a three-dimensional double helix (17). Their conclusions were based particularly on Franklin's analyses and her interpretations, but also on results of the studies performed by Levene and Chargaff. One may consider if Watson and Crick at all should be in the first line of candidates to receive the Nobel price. Levene, Chargaff and Franklin presented all the elements to describe dsDNA as a double helix three-dimensional DNA structure.

**Figure 1:**
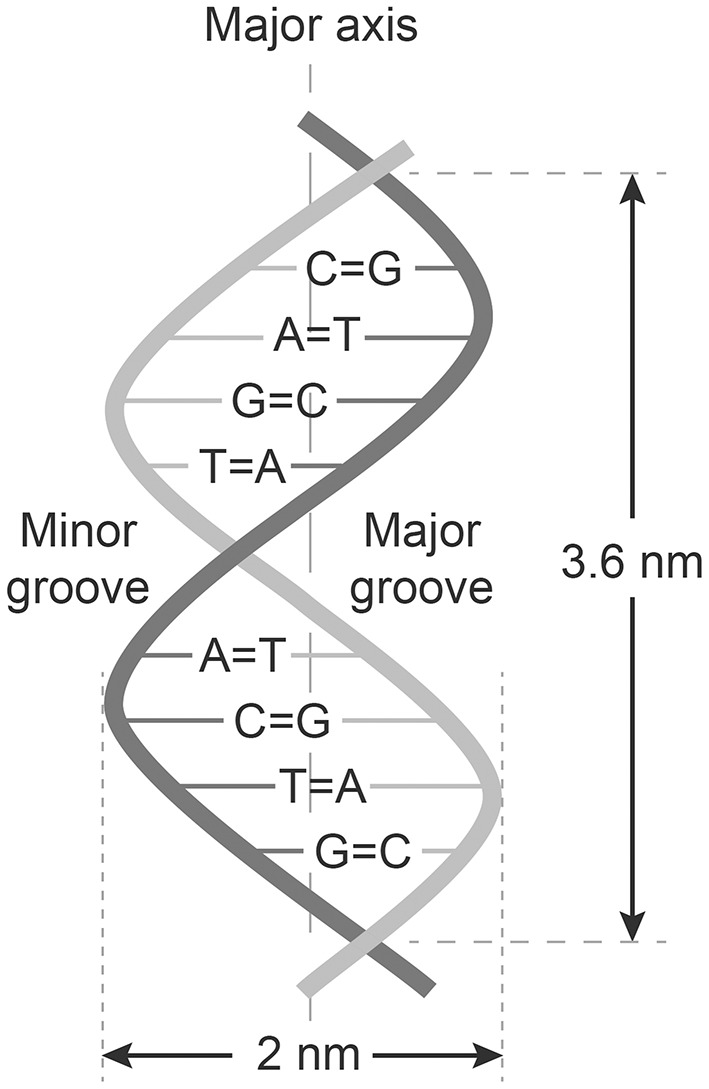
Structure of dsDNA and Chargaffs rules for a double-helix dsDNA. In this figure Chargaff's first rule demonstrates that DNA from any cell of all organisms have a 1:1 ratio (base Pair Rule) of pyrimidine and purine bases and, more specifically, that the amount of guanine is equal to cytosine and the amount of adenine is equal to thymine. This pattern is found in both strands of the DNA. The figure also demonstrates Chargaffs second rule saying that the proportion of A/T and C/G holds true for both strands.

The central work of Chargaff, Levene, and Franklin were remodeled into the paradigms now called The Chargaff's rules. These paradigms state that any DNA from cells of any species have a 1:1 ratio (base Pair Rule) of pyrimidine and purine bases. They stated that the amount of guanine equals the amount of cytosine, and that the amount of adenine equals the amount of thymine. This double helix pattern of DNA is equal in DNA from all species and provides evidence that we all evolve from the same genetical principle (see Figure 1 and Table 1).

**Table 1:** This table is a representative sample of Chargaff's et al. (13) data, taken with slightly modified table published by Bansal (18), listing the base composition of DNA from various organisms.

**Organism**	**Taxon**	**%G**	**%C**	**G/C**	**%A**	**%T**	**A/T**	**%GC**	**%AT**
Maize	*Zea*	22.8	23.2	0.98	26.8	27.2	0.99	46.1	54.0
Octopus	*Octopus*	17.6	17.6	1.00	33.2	31.6	1.05	35.2	64.8
Chicken	*Gallus*	22.0	21.6	1.02	28.0	28.4	0.99	43.7	56.4
Rat	*Rattus*	21.4	20.5	1.00	28.6	28.4	1.01	42.9	57.0
Human	*Homo*	20.7	20.0	1.04	29.3	30.0	0.98	40.7	59.3
Grasshopper	Orthoptera	20.5	20.7	0.99	29.3	29.3	1.00	41.2	58.6
Sea Urchin	Echinacea	17.7	17.3	1.02	32.8	32.1	1.02	35.0	64.9
Wheat	*Triticum*	22.7	22.8	1.00	27.3	27.1	1.01	45.5	54.4
Yeast	*Saccharomyces*	18.7	17.1	1.09	31.3	32.9	0.95	35.8	64.4
*E. coli*	*Escherichia*	26.0	25.7	1.01	24.7	23.6	1.05	51.7	48.3
φX174	*PhiX174*	23.3	21.5	1.08	24.0	31.2	0.77	44.8	55.2

*The data support both of Chargaff's rules (13, 14). An organism such as φX174 with significant variation from A/T and G/C equal to one, is indicative of single stranded DNA*.

### Chargaffs Rules (13, 14)

Chargaff demonstrated that the double helix was created and stabilized by A–T and C–G interactions. The data of his experiments were organized and summarized as Chargaff's Two Rules (see Table 1 for examples including human dsDNA):

1. The number of Adenine bases is equal to the number of Thymine bases and the number of Cytosine is equal to Guanine bases: (nA = nT; A/T = 1; nC = nG; C/G = 1), and the sum of A, T, C, G, is always 100% in the DNA double helix molecule isolated from a cell.
2. The proportion of A/T and C/G holds true for both strands.

In sum: A/T = G/C = 1.

All antibodies that bind nucleic acids characterized by the ratio in the formula 1 given above must consequently bee defined as anti-dsDNA or anti-native dsDNA antibodies.

Considering the rough methods Chargaff, co-workers and successors used to purify nuclein (DNA), the double helix must have been very robust. We know that the DNA purified for the purpose of detecting anti-dsDNA antibodies was in fact dominantly dsDNA also without further active elimination of ssDNA [(19, 20), Rekvig, unpublished observations]. Thus, the dsDNA structure described above turned out to be the target for anti-dsDNA antibodies in SLE, a statement that also may be valid for the early 1938 detection of anti-DNA antibodies in context of infections (21–23), and that complexes between them had the potential to induce inflammation in SLE-related lupus nephritis [for review see e.g., (4) and below].

## Anti-dsDNA Antibodies: How Are They Formed—and in Which Principal Clinical Contexts

To answer these questions, we have to rigorously define whether an anti-dsDNA antibody represents a response to exposed dsDNA or to a non-dsDNA/non-DNA structure by molecular mimicry (see below).

### Anti-dsDNA Antibodies: Is dsDNA a Stable Structure That May Be Immunogenic *in vivo*?

Interpretation of the structure originally called nuclein, as a derivation from Chargaffs rules, the DNA was most probably used in the first assays in the form of the canonical double helix DNA. Since the A/T and G/C ratios were stable [Table 1, and Figure 1 (13, 17)] and since they in sum did not deviate toward an overrepresentation of any of the bases that could indicate presence of ssDNA domains (Table 1), we can in retrospect conclude that the autoantibodies observed in 1957 in SLE (6–9) recognized dsDNA as a stable structure—and that they cross-reacted with dsDNA from as different species as like viral, bacteria, and mammals. DNA is present in all nucleated cells. If exposed chromatin is potentially dangerous to the body, as discussed by e.g., Darrah and Andrade (24) and by others (25–29), this may illuminate how important it is to remove chromatin from dying cells in an abrupt and silent way.

However, in individuals with anti-dsDNA antibodies and impaired clearance of cell debris including necrotic chromatin, like in SLE (30–37), this may change the situation from a controlled removal of chromatin into a condition where chromatin debris remains exposed and may be a contributor to produce and or amplify anti-dsDNA antibodies by interaction with TLR9 and to promote inflammation. This may be caused by slow removal of extra-cellular dsDNA by e.g., silencing of DNase I or blocking of DNase I activity since binding of anti-dsDNA antibodies to DNA may inhibit the effect of the endonucleases [discussed in (29)]. In this situation, externalized DNA is further targeted by anti-dsDNA/anti-chromatin antibodies, and immune complexes may be formed both *in situ* and in circulation (3, 38–40) and, as a consequence, induce serious inflammation (see below).

**Figure 2:**
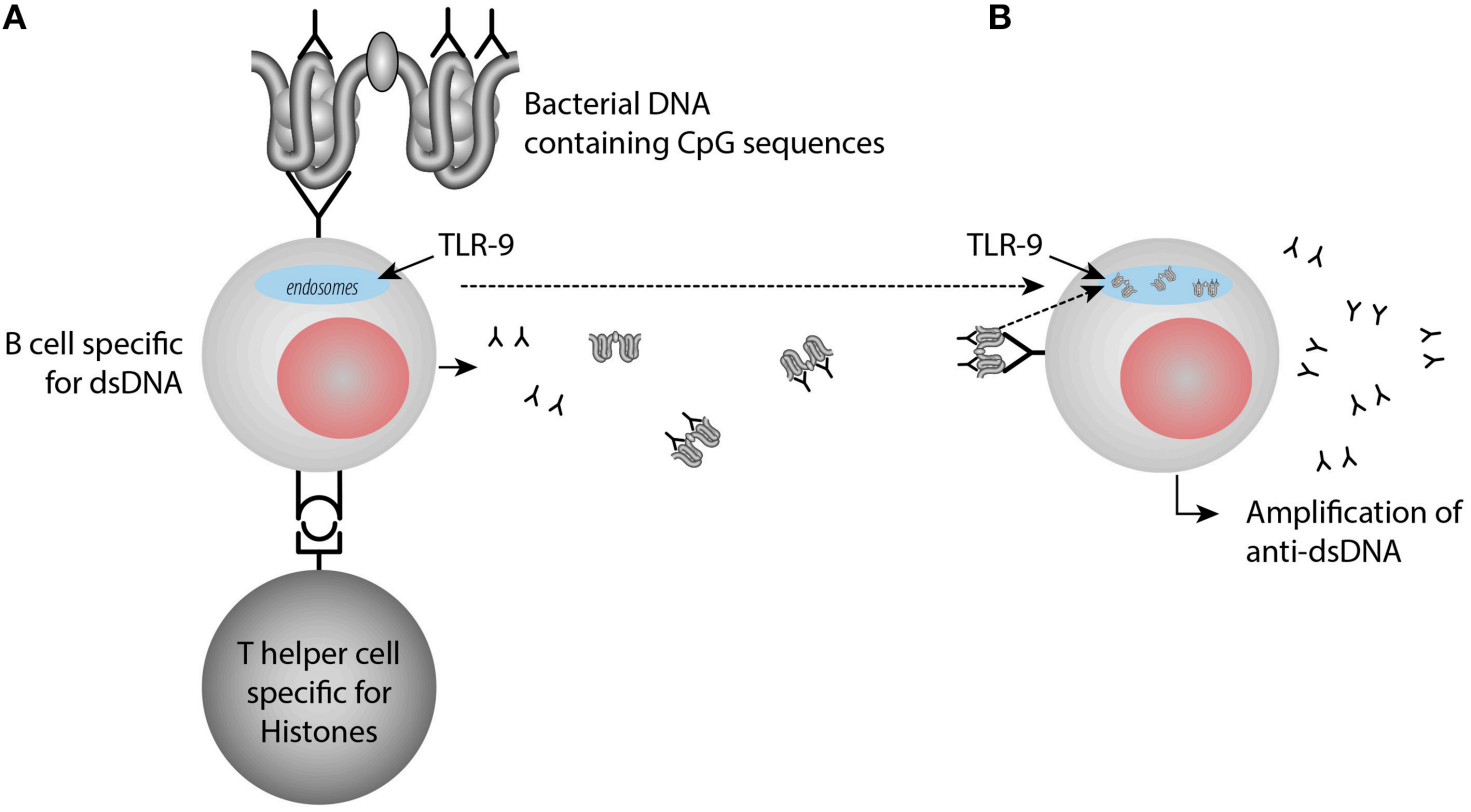
Amplification of anti-dsDNA antibody responses through activation of TLR9 by immune-complexes containing DNA-anti-DNA. In **(A)** anti-dsDNA antibodies are induced by a classical hapten-carrier complex, in which dsDNA in form of small chromatin complexes represent the hapten and histone peptides represent the carrier protein. These interactions transform B cells into anti-dsDNA antibody producing plasma cells and enter the extracellular space. Upon cell death, chromatin is degraded and removed in a fast and silent way by DNases. Anti-dsDNA antibodies bind these small chromatin fragments, make them resistant to DNase. Then they bind dsDNA through the dsDNA-specific B cell receptor and the dsDNA fragments enter into the endosomes, where TLR9 is sensing unmethylated CpG dinucleotides **(B)**. Stimulated TLR9 promotes cytokine secretion and the inflammatory response and amplification of anti-dsDNA antibodies. TLR9 promote in this way increased inflammation and amplification of anti-dsDNA antibodies upon TLR9 sensing of CpG9.

Furthermore, once anti-dsDNA antibody production is initiated (irrespective mechanism), an anti-dsDNA antibody may bind dsDNA in the extra-cellular compartment. In a normal situation, *in vivo* autologous dsDNA is rapidly and completely digested by DNases. On the other hand, anti-dsDNA antibodies may be produced and form immune complexes with the consequence that autologous DNA-containing fragments are resistant to DNases, then they may bind the DNA-specific B cell receptor and is transported into endosomes/lysosomes where TLR9 is sensing unmethylated CpG dinucleotides (41). Stimulated TLR9 acts via MYD88 and TRAF6, leading to NF-kappa-B activation, cytokine secretion and the inflammatory response (42, 43). TLR9 promote in this way increased inflammation and amplification of anti-dsDNA antibodies [(44, 45), see a model in Figure 2].

In sum, the pioneers that described nuclein (11) as dsDNA (12, 13, 17), had a substantial influence on the discovery of anti-dsDNA antibodies in an autoimmune context in 1957 (6–9) and on the potential of dsDNA and anti-dsDNA antibody complexes to drive inflammation, as we see in e.g., lupus nephritis.

### SLE: A Disease With High Rates of Infectivity and DNA-Specific Autoimmunity—Is the Latter Depending on the First?

**Figure 3:**
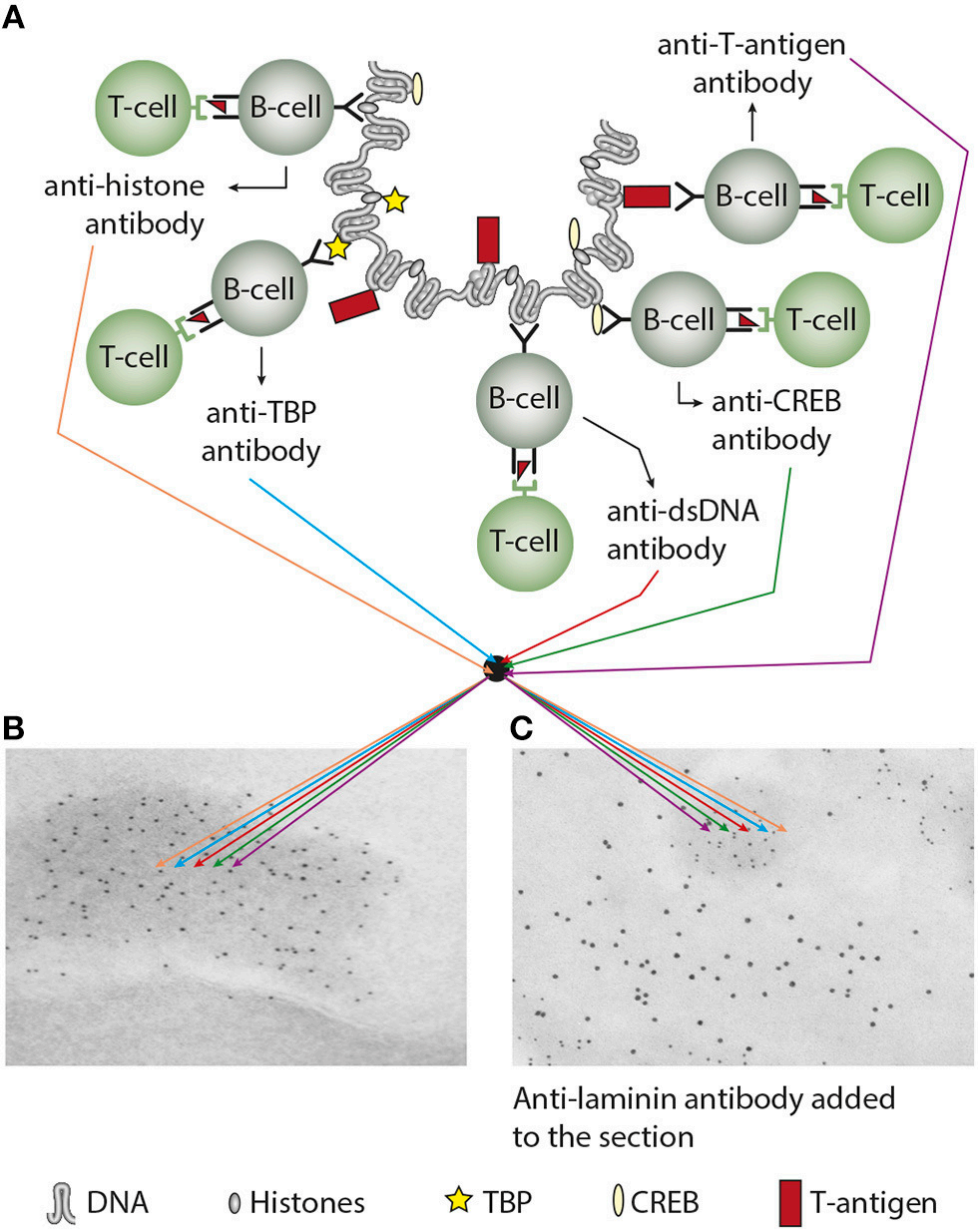
Experimental induction of anti-dsDNA antibodies and other chromatin autoantibodies by *in vivo* expression of a single viral dsDNA-binding protein. In **(A)**, Injection of normal mice with plasmids encoding wild type polyomavirus DNA-binding T antigen in context of eukaryotic promoters predictively induced production of antibodies to T antigen and significant production of antibodies to mammalian dsDNA, histones, and to certain transcription factors like TATA-binding protein (TBP) and cAMP- responsive element-binding protein (CREB). All autologous chromatin-derived ligands physically linked to T antigen can therefore be rendered immunogenic to autoimmune B cells that present peptides derived from T antigen. Therefore, concerted production of autoantibodies specific for chromatin antigens, including dsDNA and histones, is not depending on a systemic lupus erythematosus background, but may appear also in quite healthy individuals. In **(B)** the group of chromatin autoantibodies notably including anti-dsDNA antibodies target exposed chromatin in kidneys. As demonstrated by immune electron microscopy, it is evident that the autoantibodies target electron dense structure (EDS), convincingly demonstrated to constitute chromatin fragments (the left immune electron microscopy in **(B)**. These autoantibodies did not bind GBM structures or in the mesangial matrix (seen as clean membranes). However, anti-laminin antibodies added to the sections *in vitro* bound GBM (sees as 10 nm gold particle-labeled antibodies), and they did not co-localize with *in vivo*-bound anti-chromatin antibodies (seen as 5 nm gold particles) **(C)**. These data argue for the fact that anti-dsDNA/anti-chromatin antibodies bound chromatin fragments, and they did *not* bind inherent, regular membrane components. **(A)** Is copied from Rekvig (4).

Does the immune system need infection as a sine qua non-contributor to incite anti-chromatin/anti-dsDNA antibodies (see main hypothesis in Figure 3A)—and is this hypothesis a factual substantiation of the hapten-carrier system in which T cells recognize the infectious-derived chromatin-bound protein (exemplified by polyomavirus T antigen in Figure 3A) while B cells recognize hapten-like autologous chromatin structures as dsDNA, histones, transcription factors and other chromatin-associated proteins [Figure 3A (46–50), reviewed in (3, 51)].

Over so many years, we have not succeeded in understanding what the immune system recognize and act upon in context of spontaneous production of anti-dsDNA antibodies *in vivo*. Since the 1980s, studies were concerned around the following problems; what instigated anti-dsDNA antibodies, what were their targets, dsDNA or non-DNA structures (52–54), why did they correlate with disease (SLE) [(55), reviewed in (4)], how to detect them in the most appropriate way, and what make them pathogenic (56–58)? This is a concentrate of the problems being in focus over the last 50 years—*and still is*. Do cell death in context of infection, and consequent release of hetero-complexes between host chromatin and infectious-derived ligands explain the whole repertoire of chromatin antibodies in SLE (see Figure 3A), known to be overrepresented with respect to infections and to factual production of anti-dsDNA antibodies [see e.g., references (59–72)]? For example, the Epstein-Barr nuclear antigen 1 (EBNA1) (50); the C-terminal DNA-binding domain of the human papillomavirus E2 protein (46); the Fus 1 peptide derived from Trypanosoma cruzi (73); or polyomavirus large T antigen (47, 48) have all the evident and predictive potential to render dsDNA/chromatin immunogenic *in vivo* upon complex formation. Infection and autoimmunity may therefore be linked together in many situations where infections tend to be chronic or recurrent, and cell death rates are high (see Figure 3A as a model to explain linked production of different chromatin antibodies when infectious-derived proteins bind chromatin fragments). This model is not restricted to chromatin autoimmunity, but also to other autologous proteins. For example, Dong et al. demonstrated that complexes of T antigen and the tumor suppressor protein p53 terminated tolerance to p53 (74, 75).

In this sense, one idea is that infectious DNA-binding proteins and DNA/chromatin fragments are walking hand in hand in their successful promotion of chromatin autoimmunity. In this picture B cells represent the autoimmune hand while infectious protein-specific T cells represent the immune hand—and upon contact they stimulate each other and transform the B cells to be autoantibody-producing plasma cells. This has been directly demonstrated in studies were T antigen-expressing plasmids were injected in experimental animals under control of eukaryotic transcription factors (47, 48). In this context, exposed chromatin and its different molecular structures can all be *targeted* by anti-dsDNA and anti-chromatin antibodies if induced by chromatin fragment-viral DNA-binding proteins (See Figures 3A,B for a model thinking). This model says that specters of chromatin antibodies which are induced by chromatin-peptide complexes all can target exposed chromatin *in situ* and provoke serious inflammation.

### Cancer: A Group of Malignant Diseases With High Rates of Infectivity and DNA-Specific Autoimmunity—Is the Latter Depending on the First?

In this sense, cancer may represent a mirror image of autoimmunity in SLE with respect to infectivity rates and termination of tolerance for dsDNA and chromatin constituents. In line with this, anti-dsDNA antibodies are frequently detected in cancers [reviewed in (3, 4)]. In 1991, the Nobel prize winner zur Hausen suggested that most of all of human cancers worldwide are linked to viral infections, including human papillomaviruses, human T-cell leukemia viruses, hepatitis B virus, Epstein-Barr virus and polyomaviruses (76, 77). At the same time, several virus-associated cancer forms are connected with the production of autoantibodies against dsDNA [see e.g., (47, 48) reviewed in (3)]. The impact of viruses in different cancer forms, and if or how viruses influence the malignancy of tumor cells may, according to zur Hausen, need to be revised in light of new viruses that has been discovered in cancer forms since zur Hausen's data were discussed in his 1991 Science paper (76). Since cancer and SLE are largely segregated, the slight over-representation of cancer in SLE (78) does not reduce the arguments for the view that anti-dsDNA antibodies are generated independently, although possibly by similar molecular and cellular mechanisms (3) in the two different types of conditions.

In SLE, the antibodies may crossreact with and bind inherent renal antigens or chromatin fragments exposed in kidneys [(79), present study] and initiate nephritis, although the two binding profiles are principally different as one is type II and the other is type III immune mediated inflammation. On the other hand, inflammation in juxtaposition to a tumor may indicate that autoantibodies may target chromatin released from necrotic tumor cells and promote local inflammation in analogy to kidney inflammation caused by antibody-binding to chromatin exposed in glomeruli (80, 81). Implication of anti-dsDNA antibodies in tumor-associated tissue has not been directly investigated. Also in cancers, anti-dsDNA antibodies are from a principal concern not clinical epiphenomenons, although their genesis is still poorly understood (if not categorized as local infectious-driven autoantibodies as principally outlined in Figures 3A,B). One potent hypothesis may therefore be the impact in cancers of infections and anti-dsDNA antibodies that are induced by complexes of tumor-derived chromatin and DNA-binding infectious-derived peptides.

### Genesis of the Anti-dsDNA Antibody *in vivo*: Closely Linked to Infections

The role of light chain editing to abolish and control anti-dsDNA reactivity is recently discussed in SLE [see reference (4) for a brief discussion]. This type of regulation can be impaired by SLE susceptibility factors, thereby allowing DNA-specific B cells to expand in SLE [see (4) and references herein].

Till now, no clear evidence have been presented that convincingly state that anti-dsDNA antibodies are *initiated* by sole exposed autologous DNA/chromatin [(3, 51, 73), discussed in a highly relevant way back in 1994 by (82)], irrespective whether they are exposed as native or cell death-associated modified chromatin structures [discussed in (3, 29, 51)]. However, infectious-derived DNA/chromatin-binding proteins in complex with chromatin fragments can provide strong T helper cell stimuli and promote transformation of autoimmune B cells into autoantibody-producing plasma cells (3). This brings to light that the *infectivity* state characteristic of SLE or of cancers is in intimate context with (auto-)immune competent cells both physically and functionally (47, 48). This was directly hinted on already at the time of the first discovery of anti-dsDNA antibodies in 1938–1939 in patients suffering from bacterial infections (21–23, 83). Other up today examples of the link between polyomavirus infection and anti-dsDNA antibodies was shown in small children with primary polyomavirus BK infections (84). These infected children produced antibodies to polyomavirus T antigen and transiently to mammalian dsDNA. T antigen is the BK virus' transcription factor and is therefore a DNA-binding protein that in a native situation binds both viral and host cell DNA [see above, reviewed in (85)]. In this situation T antigen was assumed to serve as a T helper cell-stimulating protein presented by DNA-specific B cells, once the T cells had been primed by dendritic cells presenting T antigen-derived peptides [discussed in Figure 3, reviewed in (3)]. Thus, both along the spontaneous BK virus infection (48, 84) and as a consequence of experimental expression of T antigen *in vivo* or other infectious agents, appearance of anti-dsDNA and other anti-chromatin antibodies is a predictive outcome [(46, 47), reviewed in (51)]. Then, why do children with primary BKV that produce anti-dsDNA antibodies not develop lupus-like nephritis or dermatitis? This may be explained by absence of exposed chromatin in glomeruli and in the dermal basement membrane zone of the skin due to the transient nature of the infection. This will be further discussed below.

### Deviant Cell Death Events Promote Exposure of DNA/Chromatin—Immunogen or Target?

If exposed dsDNA in form of chromatin has the potential to induce anti-dsDNA antibodies remain as an attractive, although yet an unproven model (29). Chromatin released by cell death may be linked to aseptic inflammation, and to the role of disordered cell death processes like exposure of DNA-containing neutrophil extracellular traps (NETs), secondary necrotic chromatin, microparticles, and may be linked to reduced elimination of dead cell debris (whether of apoptotic or necrotic origin) (27, 33, 86). NETs were first observed by Brinkmann et al. (87). Still, however, their function as an assumed complex defense structure (88) is not fully resolved [see a thoughtful discussion by (89)]. On the other hand, NETS and secondary necrotic chromatin have in several studies been suspected to be involved in inflammatory processes (28, 35, 90–92), and is assumed to account for increased levels of anti-chromatin antibodies. The latter association does not, according to my understanding of the relevant literature, mean that NETS or apoptotic chromatin *induce* anti-dsDNA antibodies. This is thoroughly discussed by Gupta and Kaplan in their review (25) who reached the conclusion that “….*many of the molecules externalized through NET formation are considered to be key autoantigens and might be involved in the generation or enhancement of autoimmune responses in predisposed individuals…*..” However, they did not state that NETS had the potential to *induce* anti-dsDNA antibodies. Similarly, Pieterse and van der Vlag conclude in their study “*…it can be concluded that increased apoptosis or NETosis on its own is not sufficient to break immunological tolerance to nuclear autoantigens in SLE, and additional factors are required to turn apoptotic material or NETs into danger triggers of autoimmunity.”* (29). Still, we do not understand whether NETs, necrotic chromatin or microparticles have the potential to induce antibodies to dsDNA or to native histones, although it has been demonstrated that they may initiate antibodies against cell death-modified histones [discussed in (25)].

In support of these considerations, Radic and Dwivedi have recently published a comprehensive and critical review on controversies related to NETs, cell death and autoimmunity (93). They came to the same conclusion as presented here as they hesitate to accept that NETs promote humoral autoimmunity against native chromatin components, inclusive dsDNA. The autoimmune consequence of perturbed order of cell death and the impact on adaptive immunity is hard to comprehend. It is probably an abstraction and not proven by evidence that these processes have the potential to promote production of anti-dsDNA antibodies, although the same structures may drive innate immune-dependent inflammation in SLE (36, 90, 94). However, diminished removal of nuclear debris has been demonstrated to correlate with production of antibodies to cell death-induced structural changes of proteins in chromatin. This is in harmony with earlier observations that while histone H4 is non-immunogenic, triacetylated histone H4 is (95). Recently, Dieker et al. observed that autoantibodies against modified histone peptides in SLE patients were associated with disease activity and lupus nephritis (91).

Similarly, T cell responses to analogous modified structures do not allow us to interprete such (helper) T cells as activator of B cells and thus induce true, anti-native dsDNA autoantibodies [(26, 91, 94, 96–104), reviewed by Pieterse and van der Vlag (29)]. Data that demonstrate that deranged cell death debris can activate T and B cells specific for altered self chromatin are settled by solid experiments (26, 35, 91, 91, 92, 105). Whether antibodies or T cells against death-associated chromatin modifications have the potential to induce inflammation has not been thoroughly studied, but their recognition and binding to modified (homologous) chromatin structures exposed as NETs might well promote *in situ* formed immunocomplexes, and consequently inflammation. In harmony with these critical comments, Gordon et al. (96) demonstrated that NETs inhibition by different approaches, like genetically manipulated Nox-deficient mice, or by deletion of PADi4 or pharmacological inhibition of PAD4 activity hardly had any influence on nephritis, and NETs inhibition did not affect any aspects of nephritis, did not lead to loss of tolerance, nor to immune activation (96). Pharmacological inhibition of PAD activity did not affect the progression of nephritis into end-organ disease in inducible models of glomerulonephritis. The authors conclude that the data oppose the concept that NETs promote autoimmunity and target organ injury in SLE (96) in agreement with earlier observations by Campbell et al. (97).

Nevertheless, NETs may serve as *in situ* targets for the autoimmune responses and participate in evolution of organ injury in SLE. Thus, true anti-dsDNA antibodies may have the ability to sensitize NETs by forming immune complexes and to initiate inflammation since dsDNA in NETs may remain in their native state, and not modified during deviant cell death, as opposed to immunity to chromatin in secondary necrotic cells in which apoptotically modified autoantigens (dsDNA, high mobility group box 1 protein, apoptosis-associated chromatin modifications, e.g., histones H3-K27-me3; H2A/H4 AcK8,12, 16; and H2B-AcK12) are present (106).

Autoimmunity to dsDNA and native chromatin exists, but till now, their spontaneous appearance in a native context is still enigmatic. There is no solid evidence to say that native chromatin has immunogenic potential. However, native chromatin in complex with a DNA-binding viral protein (see above) is immunogenic because T cell tolerance, as is operative for native chromatin, is circumvented by the immunogenic infectious-derived carrier protein. There are yet no firm evidence stating that antibodies to native dsDNA are induced by perturbed cell death, although disorganized cell death may induce and *enhance* production of antibodies to chromatin-associated proteins modified in context of cell death (29, 93). Thus, although anti-dsDNA antibodies are easily detected in SLE, it is hard to explain why the antibodies materialize themselves and how they harm organs like the kidneys and skin in context of SLE (see below).

## Pathogenic Potential of Anti-dsDNA Antibodies

Isolated dsDNA is negatively charged due to solvent phase exposed phosphate groups that makes up every nucleotide that consists of pentose, nitrogenous bases, and phosphate groups (see above). This makes it unlikely that isolated dsDNA binds directly to glomerulus basement membranes (GBM) in context of lupus nephritis because GBM is overall anionic and would therefore repel dsDNA. Rather, since mammalian dsDNA is part of chromatin, consisting of histone octamers, histone H1, and a large array of other non-histone proteins with various charges, dsDNA may indirectly bind to GBM through interaction of solvent phase cationic protein tails with anionic GBM structures. This forms the basis for formation of immune complexes between anti-dsDNA antibodies and dsDNA, and deposition of the complexes *in situ* along the GBM, and in the mesangial matrix of circulating dsDNA-containing immune complexes [reviewed in (3) and (81)]. By using surface plasmon resonance, we demonstrated that isolated dsDNA did not bind collagen or laminin, while chromatin fragments bound with relatively high avidity, irrespective presence or absence of complex-bound anti-dsDNA antibodies (107, 108). These data harmonize nicely with experiments performed in the Berden laboratory, where they demonstrated that immune complexes of anti-dsDNA antibodies and nucleosomes bound in glomeruli of perfused kidneys, while highly pure anti-dsDNA antibodies did not bind (109–111). Nevertheless, these data open for two ways how chromatin may promote inflammation; either by binding anti-dsDNA antibodies to chromatin exposed *in situ*, or by binding preformed chromatin-IgG complexes to GBM.

### Anti-dsDNA Antibodies: Are They Induced by dsDNA or Non-dsDNA Structures *in vivo*?

On the other hand, anti-dsDNA antibodies have in many studies been proven to be instigated by non-DNA structures [discussed in e.g., (3, 112–114), see Table 2 for examples]. Therefore, anti-dsDNA antibodies may represent two principally different antibody populations; real anti-dsDNA antibodies induced by dsDNA, or (quasi) antibodies with potential to bind dsDNA although instigated by non-dsDNA structures. We are today not able to distinguish which is which. In context of the question if anti-dsDNA antibodies are induced by dsDNA or non-dsDNA structures *in vivo*, a logic issue would be if anti-dsDNA antibodies are *pathogenic* because they recognize dsDNA (homologous interaction) or non-dsDNA (heterologous interaction) in the kidneys.

**Table 2:** Examples of anti-dsDNA antibodies that cross-react with non-DNA structures.

**Anti-dsDNA antibody crossreact with**	**References**
α-actinin	(113)
α-actinin Laminin	(115, 116) (117)
C1q	(118)
Laminin	(119)
Nucleosomes/laminin^*^	(120)
Platelet integrin GPIIIa 49–66	(121)
Toll like receptor 4	(122)
NR2 glutamate receptor	(123)
Cell surface proteins	(124)
Ribosomal P protein	(125)
Cross-reactive anti-dsDNA antibodies (2002)	(126)
Phosphorylcholine/phospholipids	(127)
EBNA 1	(128)
Entactin Entactin^**^	(114) (129)

* *Renal eluates in this study contained several antibodies, notably with nucleosome antigens and laminin. Definitive prove for cross-reaction between laminin and nucleosomes-dsDNA was not provided*.

** *Mono-specific anti-Entactin antibodies is included to be suggested as a control of non-cross-reactive, non-dsDNA antibodies to determine if they still have nephritogenic potential (see reference (43) for details)*.

**Figure 4:**
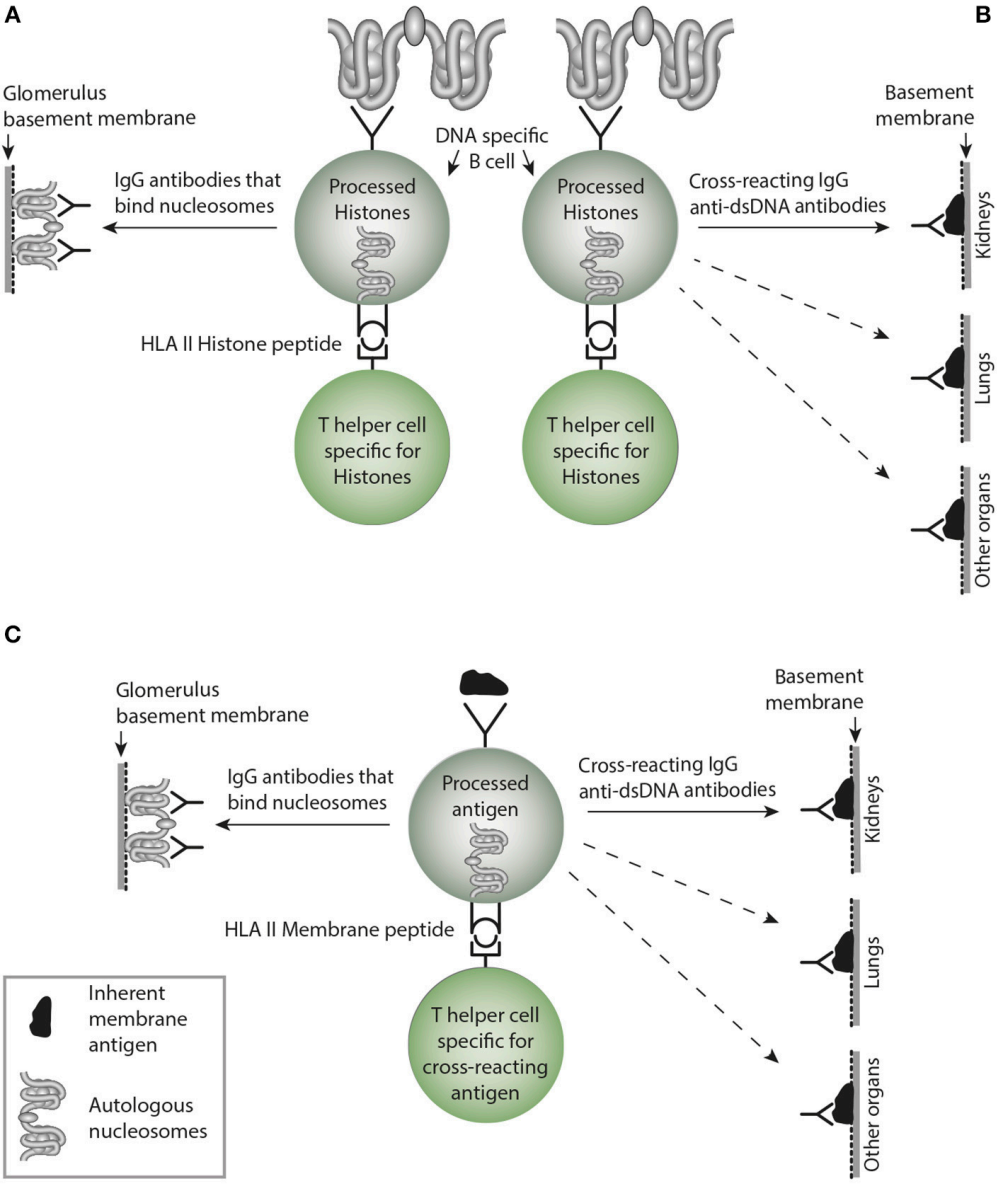
Non-cross-reacting and crossreacting IgG immune responses induced by homologous or cross-reacting antigens. In **(A)** the B cells are specific for DNA as presented in chromatin. In the left side, B cells recognize and produce antibodies that bind (dsDNA) chromatin, i.e., homologous recognition. These may target exposed chromatin and initiate lupus nephritis by a Type III immune mediated tissue inflammation. In **(B)** the B cells are specific for dsDNA, but may, however secrete cross-reacting antibodies not targeting solely dsDNA. Instead they bind non-dsDNA cross-reacting inherent glomerular basement membranes, like entactin, laminin or collagen (see text). This has not been entirely investigated, but these cross-reactive antibodies might well initiate Goodpasture analog kidney, skin or lung diseases. This has not been considered in the literature (see text). In **(C)**, the B cells are specific for a (membrane)-component and cross-react with nucleosomes. Since the IgG antibodies may recognize membrane components in e.g., lung and other organs, they inherit the nature of collagen IV-like antibodies in Goodpasture syndrome. Although many such cross-reactions have been described, they have not drawn much attention in pathophysiological contexts. More studies are needed to explore these contexts. This figure is reprinted from Rekvig (130).

Thus, anti-dsDNA antibodies may exert a pathogenic process by direct binding to inherent cross-reactive renal structures. This demonstrates that anti-dsDNA antibodies may promote two principally opposite pathogenic processes; They either bind chromatin fragments that are exposed and associated with GBM structures [denoted in this context “the chromatin model” see models in Figures 3, 4 (80)] or, they bind directly to glomerular antigens like laminin, collagen, entactin, and others by cross-reaction (denoted “the cross-reaction model,” see relevant data in Table 2, and Figure 4). Two variants of the chromatin model exist. In one; chromatin fragments are exposed in membranes and matrices due to the fact that chromatin fragments bind membranes and matrices at high affinity. If anti-dsDNA antibodies bind this form of chromatin, the immune complexes are formed *in situ* (107). In the other variant, formation of IgG-chromatin immune complexes occurs in circulation. Such pre-formed IgG- chromatin fragment immune complexes may bind primarily in the mesangial matrix and in GBM [reviewed in (3) and (4)]. The second variant is experimentally demonstrated. Injection of immunologically normal mice with highly pure anti-dsDNA monoclonal antibodies (mAbs) resulted in deposition of chromatin-fragment–IgG complexes in the mesangium and GBM (131, 132). Concentration of circulating chromatin fragments was significantly reduced after infusion of the antibodies. Similarly, if (NZB × NZW) nephritic mice were injected with pure biotinylated mAbs, these antibodies were observed in immune complex deposits observed as electron dense structures (EDS) in the mesangial matrix and in GBM (132). These data demonstrate that the experimental mAbs bound chromatin fragments *in vivo*. However, these experiments did not allow us to conclude whether they formed immune complexes *in situ* or in circulation. In line with this, circulating DNA–anti-dsDNA antibody complexes have been described (133) and discussed (134) in the context of SLE. Whether circulating complexes were associated to a certain stage of nephritis was not determined in those studies.

Combining data discussed above, mesangial nephritis, representing the early phase of lupus nephritis, may be instigated by circulating immune complexes (132), while progression of lupus nephritis into end stage organ disease is associated with silencing of the major renal endonuclease DNase I (see below). Loss of renal DNase I leaves chromatin from apoptotic cells undigested, and being retained in GBM. *In situ* formation of immune complexes by binding of circulating anti-dsDNA antibodies to the exposed chromatin fragments forms the basis for severe lupus nephritis (135). Thus, the chromatin model is in clear opposition to the cross-reactive model, and reflects the real pathogenic process of lupus nephritis (see below). It seems that we are far from reaching consensus on pathogenesis of lupus nephritis. Importantly, a proven cross-reactivity of an anti-dsDNA antibody will not provide information about which of the target antigens that binds the antibody *in vivo*.

### Why Are Anti-dsDNA Antibodies Pathogenic—and Are They All Pathogenic?

A central question is if the pure existence of anti-dsDNA antibodies is pathogenic through binding cross-reactive, obligate renal structures in SLE, or if they are epiphenomenons in absence of exposed chromatin structures (3, 4, 114, 136, 137). Thus, we have to evaluate how and why they are harmful, and in which contexts (36, 90, 94, 114, 138). This dilemma relates to the pathogenic effect irrespective whether in SLE or in other diseases like different cancer forms (see above). There is no reason to assume that an anti-dsDNA antibody produced in context of SLE differ in pathogenic impact from those produced in context of infection or cancer. There are many reasons to argue for and against these paradigms. However, these antibodies are pathogenic, but only in presence of relevant target structures. In other words; all have pathogenic potential, but they do not always transform potentiality into activity—i.e., transformation depends on whether the targets are exposed and accessible *in vivo*.

## Lupus Nephritis: Contexts and Pathogeneses

While in end 1930s, DNA without further structural distinction or knowledge was determined to be an acceptor for anti-dsDNA antibodies (21–23, 83, 139). Shortly after the presence of anti-dsDNA antibodies were confirmed, they were in 1957 described in SLE (6–9, 140). In the following text, exposed dsDNA, like in NETs, chromatin or microparticles will not be further discussed. Rather, *specificity* of nephritogenic antibodies will be the focus.

In context of the discovery of SLE-related anti-dsDNA antibodies, it was soon clear that these antibodies were associated with SLE and with lupus nephritis. This perception represented a conceptual advantage in our understanding of pathogenic processes in SLE, although Fu et al. (136) proposed that anti-dsDNA antibodies are not crucial nor necessary to cause lupus nephritis. Nevertheless, this concept derives from 5 facts: (1) DNA has been reported to bind collagen, a component of GBM (141); (2) chromatin fragments bind laminin and collagen (107); (3) the nephritogenic antibodies bind DNA (chromatin fragments) (38, 142); (4) anti-dsDNA antibodies can be purified from nephritic kidneys (38, 143, 144); (5) infusion of anti-dsDNA antibodies promote nephritis by binding glomerular structures (either GBM or exposed chromatin) in non-autoimmune mice (114, 122, 131, 132, 145, 146).

In a strict context, these facts involve recognition of DNA by antibodies linked to autoimmune inflammation in SLE, but do not necessarily provide information about which of the structures represent glomerular targets for the SLE-associated antibodies [chromatin or inherent glomerular structures, see e.g., the divergent interpretations by (130) and (40)]. The data only demonstrate that the pathogenic antibodies recognize *at least* dsDNA. As we will see, anti-dsDNA antibodies may even not by definition be denoted anti-dsDNA antibodies due to the vast number of cross-reactions/cross-stimulations with non-DNA/non-chromatin ligands or complex structures—like those in matrices and membranes (see the following discussion of this problem). Traditionally, we call this antibody family “anti-dsDNA” and/or “cross-reacting anti-dsDNA” antibodies. But these are merely biological abstractions as long as we are not able to explain their real initiators and targets *in vivo*.

## Lupus Nephritis Pathogenesis: The Need to Distinguish and Validate Pathogenic Models

From what we know today, we may be forced to define a hierarchy of antibody specificities that are involved in the genesis of lupus nephritis. This may, surprisingly, not be a concise distinction: Maybe monospecific anti-dsDNA antibody is a fiction—indicating that all antibodies are oligospecific (multiple specificities)—or at least cross-reactive (dual specificity)? We have simply not yet sufficient information about this problem [see e.g., (147, 148)]. These somewhat naïve statements cannot rule out other non-DNA lupus nephritis-associated inflammatory factors, like antibodies that are dominantly specific for glomerular structures, as collagen (2, 149), laminin (115, 150), entactin (114), or the role of T cells [see e.g., discussions in (151, 152, 152–156)]. These may be relevant candidates to understand the inflammatory genesis of lupus nephritis.

### A Hierarchy of Disparate Anti-dsDNA Antibodies Are Pathogenic in Lupus Nephritis

In this context, there is an imperative need to understand the biological and pathogenic meaning of these factual observations. Therefore, we have to dissect *in vivo*-bound antibodies and antibodies with potential to bind *in vivo*, into four categories:

- Antibodies specifically binding chromatin and DNA (51), and anti-dsDNA antibodies that may be formed as a consequence of somatic mutation, even though the reverted germ-line V regions did not show any measurable autoreactivity in the elegant study of Wellman et al. (157). Their results indicate that anti-dsDNA autoantibodies may even develop from non-autoreactive B-cells by somatic hypermutation (157);
- Antibodies that cross-react with DNA and non-DNA glomerular structures (see Table 2, for examples and corresponding references);
- Antibodies that bind native chromatin fragments but not dsDNA;
- Antibodies bound *in vivo* but have no specificity for chromatin structures, but for glomerular non-DNA structures exposed in the membranes, like entactin, laminin, and collagen [see Table 2 with relevant references, and the extensive review by (158)].

### One Pathogenic Model Implies That Anti-dsDNA Antibodies Bind Glomerulus Membrane-Associated Chromatin Fragments

Co-localization immune electron microscopy (IEM) analyses demonstrated that the electron-dense structures in mesangial matrix and in GBM were targeted *in vitro* by antibodies to dsDNA, histones and transcription factors, whereas co-localization terminal deoxynucleotidyl transferase dUTP nick end labeling (TUNEL) IEM demonstrated that these structures contained high concentrations of *in vivo*-bound IgG and TUNEL-positive DNA in both murine (159) and human (159, 160) lupus nephritis. These and similar data (135) indicate that anti-dsDNA antibodies exert their nephritogenic effect by binding to exposed chromatin fragments in glomerular membranes and the mesangial matrices (143, 160, 161), which is consistent with the fact that antibodies eluted from nephritic kidneys show specificity for chromatin fragments, histones and DNA as common denominators, although several other specificities have been detected in such eluates [see above (38, 143, 144)]. The early phase of mesangial nephritis might indeed be initiated by circulating immune complexes consisting of chromatin fragments complexed with IgG (132). Notably, by high resolution IEM we never observed antibodies bound *in vivo* to native GBM itself, nor to the mesangial matrix surrounding EDS (see Figure 3B as example, where antibodies are confined to EDS leaving GBM unstained).

#### The Role of Renal DNase I in Progressive Lupus Nephritis

We have demonstrated that progressive lupus nephritis correlates with loss of the central renal Dnase I endonuclease mRNA, and DNase I endonuclease activity. This event coincided with significantly reduced fragmentation of chromatin, leaving large chromatin fragments that accumulate *in situ* in glomeruli. If this happens in glomeruli of a person that produce anti-dsDNA antibodies, complexes of these partners (IgG anti-dsDNA antibodies and retained chromatin fragments) exert deleterious inflammatory effects on the integrity and function of the kidneys. Although not proven by solid evidence, chromatin fragments in kidneys with selectively silenced DNase I gene expression may derive from kidneys themselves, at least in progressive disease (86, 135, 162, 163).

#### Chromatin Metabolism in Kidneys in the Course of Lupus Nephritis

From both theoretical considerations and the comprehensive sets of coherent data discussed above, it is fair to conclude that glomerular extracellular chromatin fragments play a direct role in lupus nephritis, where they serve as homologous targets for anti-dsDNA/anti-chromatin antibodies. This conclusion also implies that the antibodies do not have an *a priori* nephritogenic potential in absence of chromatin. However, when chromatin is exposed in glomeruli, the antibodies are rendered nephritic (132). That is, isolated presence of either of the factors—the antibody or chromatin—remain in the body as clinical epiphenomenons! Therefore, the core nature of both murine and human lupus nephritis is pointing at an *acquired error of renal chromatin metabolism* due to silenced DNase I gene expression as a key event in disease progression [reviewed in (3, 4)].

### Another Pathogenic Model to Describe Lupus Nephritis Implies That Cross-Reactive Anti-dsDNA Antibodies Interact Directly With Glomerular Non-DNA Structures

The cross-reactive model inherits several problems that need to be described by experiments and analyses in lupus-prone mice and patients. The following questions need considerations.

#### Is the Cross-Reacting Immune Response Sustained Over Time—The Problem of Affinity Maturation?

**Figure 5:**
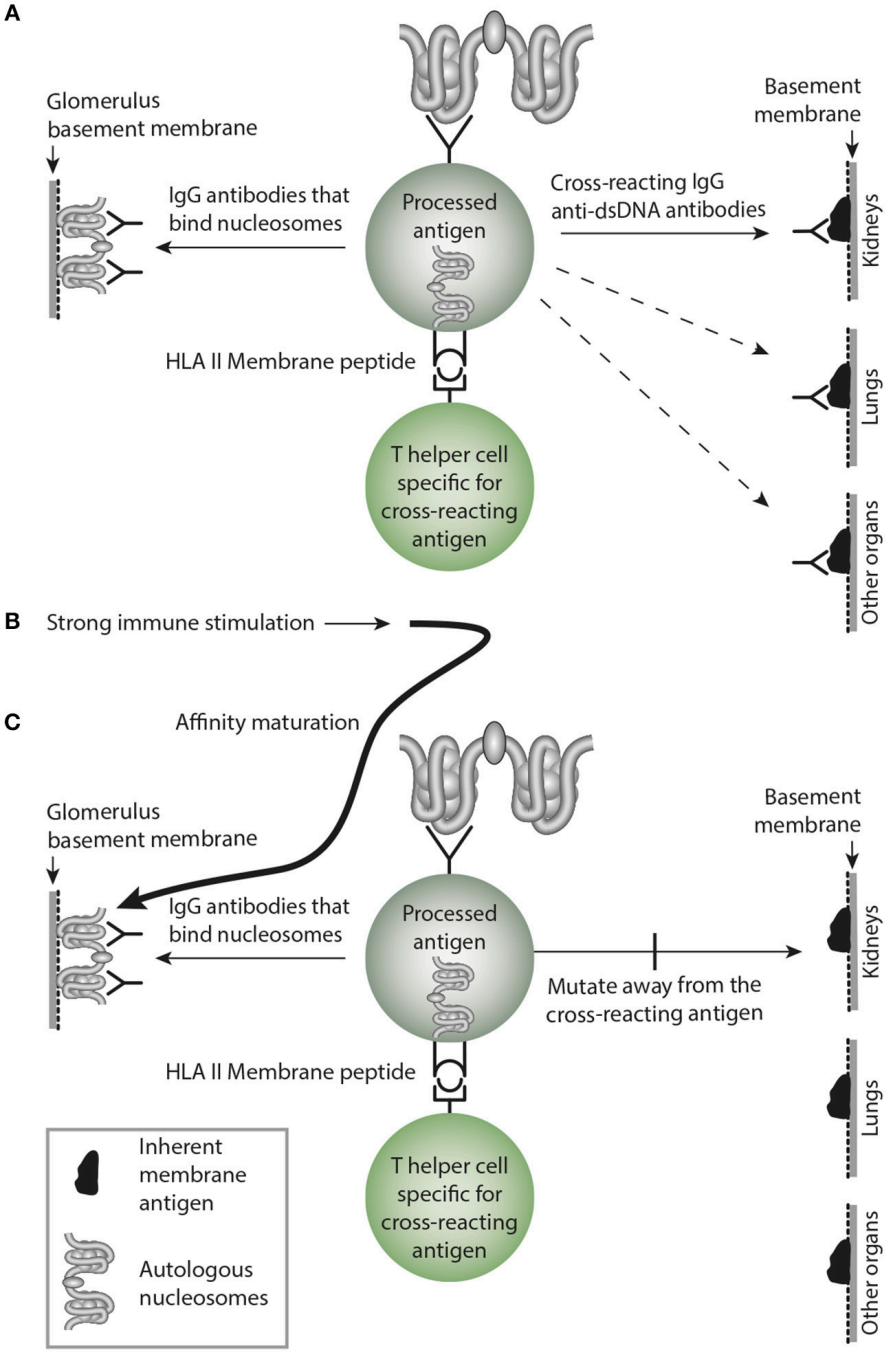
Affinity maturation may transform a cross-reacting antibody into a monospecific antibody. In **(A)** a B cell bind nucleosomes by its antigen receptor, process them and present nucleosomes-derived peptides in context of HLA class II to peptide-specific T helper cells. In this example, the B cell transform into antibody-secreting plasma cells, and the emerging cross-reacting IgG antibody recognize nucleosomes, and they cross-react with an inherent glomerular membrane antigen like e.g., laminin. Since laminin is part of membranes in different organs, the cross-reactive antibody may bind in glomeruli, lungs and in other organs, similar to the anti-collagen IV in Goodpasture Syndrome. In **(B)** a strong and possibly sustained stimulus recruits more somatically mutated IgG molecules. In **(C)** mutations are selected that promote increased affinity for the B cell antigen, while mutations diminish affinity for the cross-reacting antigen, since these antigens are not selected by nucleosomes. This is a mechanism that may transform oligospecific into mono-specific IgG antibodies. This model therefore indicates that the effect of the cross-reactive specificity may over time faint or die out.

This is a central problem in this model. Sustained immune B cell stimulation may open for a successive loss of the cross-reactive specificity while the homologous response may remain with increased avidity and titer. Considering a sustained stimulus of the dsDNA-specific B cells by dsDNA, they will increase avidity for dsDNA as a consequence of affinity mutations, and since these mutations are random, they will/may by chance mutate away from the cross-reactive specificity. Thus, over time the cross-reactive specificity may slowly die out due to sense mutations for the immunogen (dsDNA), and non-sense mutation for the crossreacting specificity that inevitably will die out (see model thinking as presented in Figure 5).

#### Is the Avidity for a Cross-Reactive Antibody Similar for Both Ligands or Will the Highest Avidity Direct the Antibody to That Antigen?

When we started our studies on the pathogenesis on lupus nephritis, we foresaw this problem. Therefore, we developed highly sensitive electron microscopy (EM) variants that with relatively high precision could determine the nature of the glomerular targets for nephritogenic antibodies. This included transmission EM and IEM to trace binding of antibodies *in vivo*, co-localization IEM, where we added different experimental antibodies to the renal sections, like antibodies to dsDNA, histones, transcription factors and GBM ligands like laminin, in order to analyze which of the added antibodies co-localized with *in vivo*-bound IgG. In addition, we analyzed loci of *in vivo* bound antibodies by TUNEL-colocalized IEM where we observed that TUNEL-positive DNA co-localized with *in vivo*-bound IgG. All our results were consistent and demonstrated that IgGs that bound *in vivo* were exclusively seen in EM as part of electron dense structures (see details in Figure 3B). No binding to podocytes or to regular GBM structures were observed (79). The same was true when we translated these experimental analyses to human lupus nephritis (79, 159).

#### Will Antibodies Mono-Specific for a Non-DNA Cross-Reacting Antigen Bind in Glomeruli?

This question—and obvious deviating experiments—is in fact neglected in the literature in this context. We know that many anti-dsDNA antibodies cross-react with a large panel of non-dsDNA structures [See Table 2, and e.g., (112)]. By injecting cross-reacting (dsDNA-X) and non-cross-reacting non-dsDNA (X) antibodies into mice, may solve if one—or both specificities contribute to lupus nephritis.

#### Similarly, If Crossreaction of Anti-dsDNA Antibodies With Renal Antigens Is Instrumental in Initiating Lupus Nephritis, Then Why Does the Disease Start in the Mesangial Matrix?

This is exactly what we observe in the BW mouse after injection of purified anti-dsDNA antibodies (135), and linked to loss of renal DNaseI endonuclease, the disease expanded from mesangial nephritis to membranoproliferative nephritis with deposits of the antibodies in GBM where they co-localize with chromatin fragments. If the antibodies bound *in vivo* crossreacted with e.g., laminin or entactin, we expected they should bind simultaneously in the mesangium and GBM.

#### Are Cross-Reactive Antibodies Eluted From Nephritic Kidneys?

In search of the biological meaning of cross-reacting antibodies as essential in lupus nephritis, there are so far too few systematic analyses addressed to solve this problem. One clear exemption is the study of Deocharan et al. They analyzed anti-dsDNA antibodies that crossreacted with α-actinin and observed strong antibody activity in renal eluates (113). However, it is difficult from such observations to determine if the antibodies bound exposed chromatin or exposed α-actinin. More important, it would have been of strong interest if control injection with a non-cross-reacting (non-dsDNA) counterpart was performed. If they could be rescued from kidneys by elution, it would have been easier to make stronger conclusion on the impact of assumed pathogenic cross-reactive antibodies.

## Concluding Remarks

This study discusses two central problems: Are antibodies binding dsDNA really anti-dsDNA antibodies, or do they recognize dsDNA after being instigated by a non-dsDNA (cross-reacting) antigen. Secondly, and in line with the first problem, are these antibodies nephritic because they bind chromatin exposed on glomerular membranes or are they nephritic because they recognize inherent glomerular membrane structures. These two models—the chromatin model and the cross-reactive model—are still not fully understood, have not been agreed on, and are still promoting controversies. Yet, the discussions and contradictions aimed to describe the pathogenesis of lupus nephritis characterize the contemporary situation. Thus, we still lack a coordinated and open minded approach to obtain a general and evidence-based perspective by not taking all aspects of the syndrome SLE into account. This can only be achieved by concentrating on the biological and pathophysiological interactions between its different disease-promoting elements. We need a framework in which dissection of published data generated by experts in different fields like immunology, pathology, immunopathology, and experimental animal research can be combined and confronted with each other simply in order to determine what we agree on (is the anti-dsDNA antibody important?), what must be done (study the impact of the other side of the cross-reacting anti-dsDNA antibody), and what the best strategy forward must be (to collaborate between the different schools of hypotheses). Whatever its nature and origin might be, anti-dsDNA antibodies are a strange and challenging phenomenon—so is lupus nephritis and SLE also. And do not forget the role of T cells in lupus nephritis! As a conclusion for now, we are producing increasing numbers of puzzle pieces connected to the eponym SLE. We are not, however, halting and concentrating on organizing the picture that may tell us why the puzzle pieces belong to each other. New phenomenons are not needed if we do not put them into a context leading to our understanding of SLE and how to treat it.

## Disclosure

The author declares he has no competing interests, or other interests that might be perceived to influence the results and discussions reported in this manuscript.

## Ethics Statement

The present manuscript is a review on murine and human SLE and lupus nephritis. All data are taken from original studies approved by relevant ethical committees.

## Author Contributions

The author confirms being the sole contributor of this work and has approved it for publication.

### Conflict of Interest Statement

The author declares that the research was conducted in the absence of any commercial or financial relationships that could be construed as a potential conflict of interest.
